# Development of a Mobile App for Occupational Stress Screening Among Female Workers: Protocol for an Exploratory Sequential Design Study

**DOI:** 10.2196/55874

**Published:** 2024-11-14

**Authors:** Dini Widianti, Zwasta Pribadi Mahardhika, Robiana Modjo

**Affiliations:** 1 Faculty of Medicine YARSI University Jakarta Pusat Indonesia; 2 Faculty of Public Health University of Indonesia Depok Indonesia

**Keywords:** mobile app, study protocol, occupational stress, female workers, stress, screening, worker, app, safety hazards, technological innovation, ergonomic, psychological hazards, mobile health, digital platform, algorithm

## Abstract

**Background:**

Occupational safety hazards include physical, chemical, ergonomic, biological, and psychological hazards. Technological innovation in screening for occupational stress, especially among female workers, is needed to improve their health and productivity.

**Objective:**

This research is being conducted to obtain a prediction model of work stress through a questionnaire instrument that includes stressors and symptoms based on the transactional model, as well as measurement of work stress through a mobile app that can be used anywhere.

**Methods:**

The research is conducted in 3 stages: qualitative research, quantitative research (cross-sectional), and mobile app development. Data were collected from companies located in Jakarta, Indonesia. The sample was chosen based on purposive sampling. For the quantitative research (n=430), logistic regression analysis was used.

**Results:**

We are developing a work stress screening instrument for female workers, which includes stressors and symptoms based on the transactional model, in the form of a digital platform so that female workers can undertake the examination anywhere without interfering with working hours or home duties. This research was funded in January 2024 and qualitative data collection began in February 2024. Quantitative data were obtained in March 2024; the number of respondents in the qualitative stage was 6, and in the quantitative stage it was 430. The work stress screening app is in the development stage and will be launched at the same time as the data collection is performed so we can examine the respondents’ perspectives on the use of the app.

**Conclusions:**

This study analyzes the prediction of work stress to help female workers screen for work stress. Workers who are detected as experiencing work stress will be educated using an algorithm programmed in the app.

**International Registered Report Identifier (IRRID):**

PRR1-10.2196/55874

## Introduction

The promotion of safety and preventative health is done through the creation and implementation of national policies, systems, and programs on occupational safety and health; a national system should implement appropriate measures to protect all workers, especially workers in high-risk sectors and vulnerable groups, such as informal-economy workers, migrant workers, and young workers or women. In designing national systems, the prevention recommendations should use a gender-sensitive approach to protect both women and men [1]. Stress is an external or internal stimulus that produces a compensatory biological response and can trigger or aggravate many diseases or pathological conditions [2]. Occupational stress has a significant adverse effect on workers’ well-being, productivity, and performance and is becoming a major concern for both individual companies and the overall economy [3]. Female workers have special needs, and this has been the basis for the emergence of special regulations for them, such as the right to menstrual leave, the right to miscarriage leave, the right to maternity costs, the right to breastfeed after childbirth, and the right to special facilities [4]. Stress theory has evolved, but fundamentally, it can be categorized into three approaches: (1) the stimulus (stressor) stress model, (2) the response stress model, and (3) the transactional model [3,4]. The use of apps for occupational stress screening can help workers screen on the go, thereby reducing the impact on professional workload, increasing cost-effectiveness, and encouraging tactics for self-examination [5]. Progress in the field of occupational stress is based on attention to the issue of measuring occupational stress [6]. Past research has observed that multiple roles, lack of career advancement, discrimination, and stereotyping are factors that create stress among women, confirmed that women report higher levels of stress than men, and concluded that female entrepreneurs feel more stress than their male counterparts [7]. The inability to spend sufficient time for family and friends, child care, and children’s education are considered highly stressful by both male and female entrepreneurs. It has been observed in Nigeria that obtaining annual leave or help from colleagues as coping strategies for organizational and personal stress are most widely used by female employees of commercial banks [8,9]. In some workplaces, women are still considered supplementary breadwinners for the family, so they are given lower wages than men, even when the workload and type of work are the same [9]. The annual cost of mental health problems is estimated to be more than €135 billion (US $145 billion) in the European Union (5% of gross domestic product), between US $150 billion and US $300 billion in the United States, and CAD $50 billion (US $35.9 billion) in Canada [10]. Occupational stress is a significant problem in occupational health, involving enormous costs for workers, employers, and governments. The European Agency for Occupational Safety and Health has recorded that more than US $550 million is lost each year in the United States because of workdays being missed due to stress. The Labor Force Survey also reported a US $11.3 million loss due to missed workdays among 487,000 employees in the United Kingdom in 2013 and 2014 [11].

This situation can be prevented by conducting mental health–related screening and comprehensive occupational stress screening among female workers. There is no existing occupational stress screening instrument specifically designed for female workers, even though they have a higher risk of experiencing occupational stress and mental health problems. The World Health Organization recommends strengthening mental health services by using mobile platforms so that the full spectrum of mental health needs can be met through a network of accessible, affordable, and high-quality community-based services and support [12]. Mobile health is the use of mobile devices to reinforce, sustain, and support public health interests, and it has become a widespread platform for the promotion and continuity of individual-led self-care, enhancing person-centered care and driving the advancement of health literacy to positively change people’s views on screening or diagnosis [5].

Our research questions are as follows: What are the predictors of work stress for female workers? What is a valid and reliable occupational stress screening instrument for female workers? and What mobile app is best suited for occupational stress screening?

Out objective is to develop a mobile app–based occupational stress prediction model for female workers. Specifically, our objectives are to (1) identify predictors of work stress for female workers, (2) develop a valid and reliable work stress screening instrument for female workers, and (3) create a work stress screening mobile app.

## Methods

### Overview

**Figure 1:**
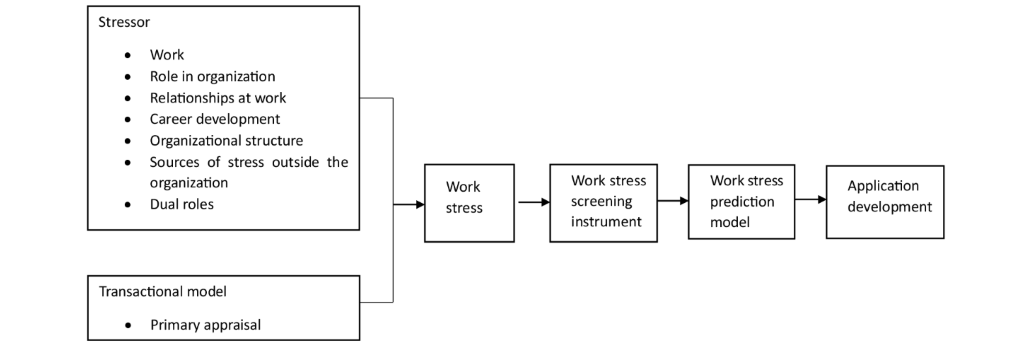
Conceptual framework.

**Figure 2:**
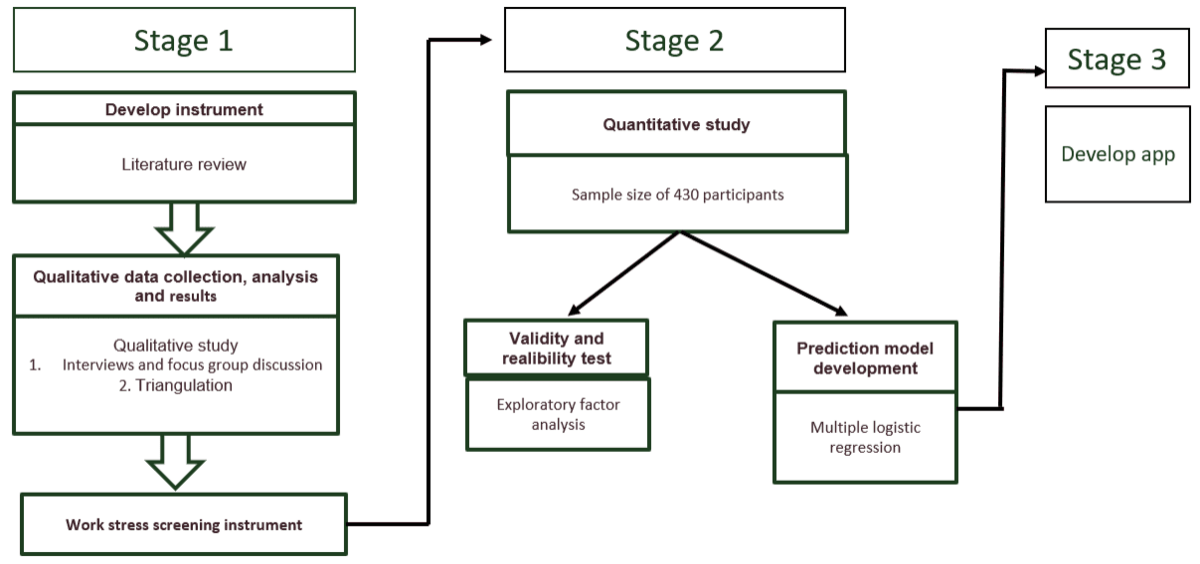
Research stages.

The conceptual framework of this research is shown in Figure 1, which shows the phases of the research. The objectives of these phases were achieved using operational research, which was conducted sequentially in 3 stages (Figure 2). The participants involved in the qualitative research worked at various levels of health care. Participants were selected with various characteristics to enrich the findings. The workers involved were diverse, representing the sources of the participants. Data validation was conducted using theoretical triangulation [13]. This sampling design was expected to be representative of female workers in Jakarta, Indonesia. Female workers were involved in stages 1 and 2 in the hope that they could provide additional information about work stressors.

### Operational Definition

Work stress assessment includes work (workload, work schedule, working conditions, work environment), roles in the organization (role conflict and ambiguity, role vagueness), relationships at work (relationships between colleagues or superiors), career development (job promotion, lack of job security), organizational structure (lack of participation in making decisions), sources of stress outside the organization (family problems, financial difficulties), having multiple roles (time-based conflict, strain-based conflict, behavior-based conflict), the transactional model (primary appraisal), and symptoms of work stress (physiological symptoms, psychological symptoms, behavioral symptoms).

### The Qualitative Phase

For data collection, inductive (codes drawn from semistructured personal interviews) and deductive (codes drawn from the literature review) methods will be used. Semistructured personal interviews will be conducted with female workers. An interview guide will be used for the interviews. This guide will be developed in consultation with experts in qualitative research and occupational stress. Each interview will open by asking open-ended questions about work stress, the effects of work, and the role of female workers as wives or mothers in the household. Questions will be asked based on the answers to the first question and other questions also based on the interview guidelines; if needed, the researcher will use probing questions, such as “What do you mean by this?” “Can you give a more detailed explanation?” and “What do you feel about this topic?” In addition, at the end of each interview, the participant will be allowed to talk about any points that were missed. All interviews will be recorded and transcribed verbatim; during the interview, respondents’ nonverbal responses, such as tone, silences, emphasis, crying, and sighing, will also be documented [14].

The researcher interviewed an informant by visiting the workplace where the informant worked to seek approval and set the time and place of the interview.

#### Data Analysis

Processing of data obtained through the in-depth interviews was done through the following steps: (1) collecting data obtained from informants through interviews; (2) performing data transcription; (3) categorizing data to facilitate data grouping and data interpretation in the form of a matrix; (4) presenting a summary of data from in-depth interviews in the matrix; and (5) conducting data analysis using content analysis techniques, namely analyzing the primary data to identify the results and compare them with existing theories (this will be used to produce a collection of main items for the instrument).

#### Sample Size and Sampling Strategy

The research was conducted in the Daerah Khusus Ibukota (DKI) region in Jakarta from November 2023 to February 2024. The qualitative part of this research collected information from several related sources, including female workers, the DKI Jakarta Manpower and Transmigration Office, Badan Penyelenggara Jaminan Sosial (BPJS) Employment (a social security organizing agency), Apindo (the Indonesian Employers Association), trade unions and labor unions, and company stakeholders. The key participants were female workers.

Participants were selected who met the inclusion criteria of being older than 17 years; having a work history (divided into groups for 0-2 years or >2 years); having completed some form of education, either primary school, high school, or college; being unmarried or married; and either not having children or having children younger than 5 years.

The female workers in the sample were white collar workers, that is, those with job descriptions or tasks that are predominantly in an office, cubicle, or other administrative setting. This group of workers uses minimal physical exertion and rarely performs manual labor.

### The Quantitative Phase

The focus of this phase is on assessing the psychometric properties of the female worker screening questionnaire, that is, its face, content, construct, divergent, and convergent validity, as well as its reliability [12,13]. The objectives of the quantitative research included assisting researchers in making decisions and obtaining results that could be used to make appropriate theoretical predictions and develop mathematical models; thus, this research not only uses theory or literature but also measurement as a very important aspect [15].

#### Validity and Reliability Test

The analysis at this stage included a factor analysis, which is used to validate new questionnaires created to measure abstract factors [13,14]. In this study, exploratory factor analysis was used to find the primary constructs or dimensions to improve the validation of the work stress screening instrument for female workers that had been developed and tested in the previous stage.

The first process we undertook was to examine the correlation between items; if there was a correlation between items of 0.6-0.7, it indicated that there was multicollinearity between the 2 items. If this was found, then one of the items had to be eliminated. The second process we undertook was to determine the adequacy of the sample size with the Kaiser-Meyer-Olkin (KMO) measure and the Bartlett test value. A sample is said to be adequate if the KMO measure value is >0.50 (0.50-0.70=moderate; 0.71-0.80=good; 0.81-0.90=very good; >0.90=superior). The Bartlett test significance value is expected to be <.05 if there is a meaningful correlation between assessment items. If the KMO and Bartlett tests meet the above requirements, then an interpretation of factor analysis can be performed [16]. The following process was used to determine the number of dimensions that could be formed from the assessment items. In other words, all items from the tested questions and statements could be grouped into factors or dimensions through having eigenvalues >1 or by using scree plots. The total variation in work stress scores that can be explained through the dimensions formed is expected to be ≥0.6 or 60%. After the number of dimensions was successfully determined, we determined the loading factor (ie, correlation between the assessment item and the dimension), which was 0.4 [5]. Finally, assessment items were included in the work stress screening instrument that had an interitem correlation of 0.3-0.9, a minimum correlation of 0.4 with each dimension, and a Cronbach α (internal consistency) of at least 0.6 for each dimension [17]. The work stress scores analyzed in relation to stressors, the transactional model, and symptoms comprised the final work stress screening instrument scores from the factor analysis.

#### Sample Size and Sampling Strategy

The variables to be examined quantitatively were elements of job stress, representing a type of primary data. Quantitative data collection was done using instruments that were developed for this study. The type of data collected was primary data. The data were distributed using Google Forms to female workers. The sample size was calculated based on the rule of thumb sample size for factor analysis, which is 20 subjects per variable [18].

The sample size was set at 390 with an anticipated dropout rate of 10%; thus, the final sample size for quantitative research was 429, rounded up to 430.

The sample was selected using purposive sampling because there were no data on types of businesses or their company names. Respondents were selected based on their employment by companies that had female workers and were willing to participate in the research. The inclusion criteria for the research locations were that they be the premises of companies that had more than 50% female workers and used mass production techniques. The inclusion criteria for research respondents were that they be physically and mentally healthy and aged at least 17 years. The exclusion criteria for research respondents were that they did not work at the workplace, were challenging to communicate with, or were sick or recovering from treatment. The female workers in the sample were all white collar workers.

#### Statistical Data Analysis

Univariate analysis aims to create a detailed picture for data from each variable. For categorical variables, data are presented in the form of percentage values. For numeric variables, data are presented in the form of means, medians, and modes. In addition, values for variation are also presented, namely SD, SE, and minimum and maximum values of the data.

We used a bivariate analysis to show differences in the proportion of work stress in female workers. The statistical test used was the *χ*^2^ test. The basis of the *χ*^2^ test is a comparison of the observed frequency with the expected frequency. The significance level used was 95%, with a significance value of 5%.

The criteria for relationships were based on the resulting *P* values (ie, probability) for the selected significance values, with the following criteria: if the *P* value was <.05, it was considered significant, and we considered there to be a difference in the proportion (ie, the null hypothesis was rejected), and if the *P* value was <.05, then we considered there to be no difference in the proportion (ie, the null hypothesis was not rejected).

This analysis aimed to determine the variables that most affected work stress in female workers. The test performed was multiple logistic regression. This analysis is a mathematical-model approach that aims to analyze the relationship between one or more independent variables with a dichotomous/binary categorical dependent variable.

In the first stage, bivariate selection is carried out to obtain variables that are included in multivariate modeling. In this bivariate selection stage, the independent variables that are eligible for selection are those that have a *P* value <.25, as seen in the omnibus test value. The next stage is multivariate selection, which is carried out in stages by removing the variables with the most significant *P* values; variables with *P*>.05 are removed. If the excluded variables result in a significant change in the odds ratios of the remaining variables (change >10%), then the variable is re-entered into the model.

In the next stage, an interaction test is conducted on variables that are suspected to have substantial interactions. If the interaction test results in a *P* value <.05, then the variable is included in the model.

### Mobile App Development

The development of a work stress screening app for female workers is being conducted in stages, consisting of requirement planning, analysis, design, prototyping, and implementation, until there are no inputs or complaints based on system visualization. This prototyping method was chosen because it provides facilities for developers and users to interact with each other during the creation process so that developers can easily model the software to be created [19].

#### Planning Stage

At this stage, the IT team determines requirements to identify the needs of the app users and determine which needs can be implemented into the app. The method used is interviews with researchers regarding needs and expectations; these will be analyzed to determine the features of the app.

#### Analysis Stage

At this stage, 2 types of system requirements are collected and analyzed, namely functional requirements and nonfunctional requirements.

#### Design Stage

In this stage, we not only used unified modeling language (UML) to create use cases and activity diagrams, we also carried out a design process based on the design thinking method. Design thinking is generally defined as an analytical and creative process that involves a person experimenting, prototyping models, collecting feedback, and redesigning [20].

#### System Prototyping Stage

At this stage, the authors will create a prototype based on the results of the design stage that can perform interactions to be tested and presented to the researchers.

#### Implementation Stage

At this stage, the prototype will be implemented as an app with similar functions and features as the prototype. This will then be tested by researchers to determine whether it is appropriate and answers the problem or needs evaluation; improvement of the app will be undertaken.

#### Testing Stage

At this stage, the system that will be implemented will need to be tested to see whether it aligns with what is planned and expected. Testing will be done using the black box method and will be conducted among female workers. The testing of the system will aim to determine the extent to which the system can meet user needs and fulfill all requirements. Black box testing will be carried out based on app details such as app appearance, functions of the app, and the suitability of the function flow for the desired business process [21].

**Figure 3:**
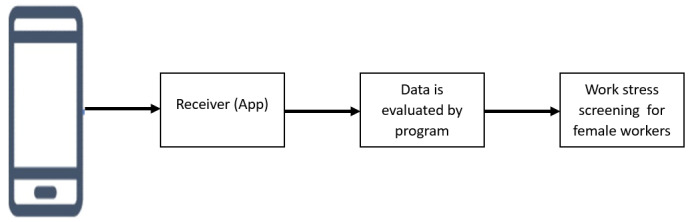
Mobile app for work stress prediction among female workers.

The app being developed will be called DINI Sehat Paripurna (Detection and Efforts to Normalize Immunity of Female Workers) and will be available for free download from the Android Play Store. It will be a work stress app (Figure 3) that will be tested by IT experts and female workers, who will assess the functionality and content of the system. The objective of this app will be to predict work stress in female workers and provide education. Female workers will answer the questionnaire in the app, and after completing all the questions, they will receive results indicating mild, moderate, or severe work stress (Figure 3).

### Ethical Considerations

#### Overview

The researchers will collect data from female workers using an online questionnaire. Data collection aimed to screen for work stress in female workers. There were no right or wrong answers in data collection, so respondents could provide answers according to their actual conditions. This method is useful to determine work stress in female workers based on the conditions identified, and allows efforts to be made to protect the workers’ mental health.

#### Right to Resign and Compensation

Participation in this study was voluntary, and respondents had the right to withdraw at any time without adverse consequences to the respondents. Respondents who were willing to continue received a payment of IDR 25,000 (US $1.58).

#### Data Confidentiality

This study was performed according to research rules that included keeping the identities of the respondents confidential and using the data collected only for scientific activities. All respondents participated in filling out the questionnaire voluntarily.

#### Ethics Approval

At the time of publication, we were in the process of obtaining ethics approval from the Ethics Commission for Research and Community Health Services, Faculty of Public Health, University of Indonesia.

## Results

This research was funded in January 2024 and qualitative data collection began in February 2024. Quantitative data were obtained in March 2024; the number of respondents in the qualitative stage was 6, and in the quantitative stage it was 430. The work stress screening app is in the development stage and will be launched at the same time as the data collection is performed so we can examine the respondents’ perspectives on the use of the app.

## Discussion

### Overview

As an essential part of psychological-related research activities among workers, the assessment of occupational stress requires the use of appropriate instruments. The occupational stress questionnaire described here will be used to collect data on female workers’ occupational stress and measure their level of occupational stress.

The questionnaire in this exploratory sequential study will be developed based on a definition of work stress that centers the perspective of female workers. Then, the psychometric properties of the questionnaire will be assessed. Given the lack of comprehensive work stress questionnaires for female workers, the development of a mobile app for work stress screening among female workers can be seen as an essential step in assessing and promoting the mental health of this population. The strengths of this study are the exploratory sequential design, the use of sampling among various stakeholders, and the relatively large sample size.

### Limitations

A limitation of this study is the difficulty in obtaining data on companies that have female workers; the sample of workers was obtained only from companies that were willing to participate in this study. Furthermore, the analysis uses structural equation modeling to determine simultaneous linear relationships between work stress and variables that cannot be measured directly (ie, latent variables).

### Conclusion

This research will screen for work stress among female workers with a method that can be used anywhere, based on a mobile app. This research will be useful for female workers, relevant stakeholders, policy makers, and academics.

## Abbreviations

Apindo: Asosiasi Pengusaha Indonesia
BPJS: Badan Penyelenggara Jaminan Sosial
DKI: Daerah Khusus Ibukota
KMO: Kaiser-Meyer-Olkin
UML: unified modeling language
WHO: World Health Organization

## Data Availability

The datasets generated and analyzed during this study are available from the corresponding author on reasonable request.
